# Coming Together: R&D and Children’s Entertainment Company in Designing APPs for Learning Early Math

**DOI:** 10.3389/fpsyg.2018.02751

**Published:** 2019-01-24

**Authors:** Carlos Mera, Gonzalo Ruiz, Manuel Aguilar, Estíbaliz Aragón, Cándida Delgado, Inmaculada Menacho, Esperanza Marchena, Manuel García Sedeño, Jose I. Navarro

**Affiliations:** Department of Psychology, University of Cádiz, Cádiz, Spain

**Keywords:** APPs, early math, research transfer, spin-off, gamification

## Abstract

Information and Communication Technologies (ICT) have an increasing influence on the way we interact, learn, and live. The increase in teaching and learning methodologies that are mediated by ICT in the field of education and in the domestic settings encourages the design of new effective technological tools, supported by scientific research and development to improve student learning. The challenge psychology is facing in the education field is to promote those technologies and make them available to the education community. Technologies also would produce attractive items for users and realistic commercial issues for businesses. This also allows an effective transfer for scientific work, providing visibility to Research and Development. In this context, the main aim of the article is to describe the process to get an agreement between Babyradio (a children’s entertainment company: https://babyradio.es/) and our research team, starting a collaborative work between two groups of people (Babyradio’s technical designer and Psychologist-Engineers software designer), in order to create several educative applications (APPs) in the field of early mathematics cognition. The institutional framework of the relationship of the R&D project and a children’s entertainment company is described. The article also focuses on experience in Psychology, Technological Innovation, and Entrepreneurship. In considering the efficiency of the agreement, we present different APPs designed for tablets and smartphone devices, adapted to the different operating systems (IOS, Android, Windows). APPs are designed to instill the cognitive fundamentals associated with early math learning for students aged 4 to 7 years. The study developed after this babyradio-university enterprise agreement contributes to the development of mathematics skills in children, aged 4–7 years, so that they can successfully meet the mathematics school requirements; it also contributes to encouraging a more positive attitude toward mathematics. This study also suggests how the education system and software and educational content developers’ companies would manage verified instructional APPs, with a more realistic commercial perspective.

## Introduction

Mathematical learning is an essential tool for school success and also has an important impact on personal adaptation to everyday life. Teaching mathematics in schools has received different criticisms from experts. They believe the method should be substantially modified in order to reduce the current failure in this matter (Martínez Montero, 2017). Significant efforts have been made in the last decade to change this tendency, although results have been very dissimilar. Methods such as “open calculation based on numbers” (ABN), have allowed some countries to substantially modify the teaching-learning process of mathematics starting with the pre-primary school levels (Aragón et al., 2017). But it is necessary to carry out studies that confirm advantages of this new type of methodology (Cerda et al., 2018).

Information and Communication Technologies (ICT) have an increasing influence on the way of interaction, learning and life. ICT are being used in different contexts to teach mathematics at different school levels (Kushwaha and Singhal, 2017). These new technologies are widely used in everyday life by children. The educational system must know how to take advantage of ICT and neutralize the disadvantages.

One of the weaknesses of the use of ICT in mathematics education is related to content design. Although there are many companies dedicated to making math educational materials, a line of research and development is necessary to guarantee the reliability and validity of the contents designed. In this area, there is still a very wide margin of improvement that must be accomplished.

The business awareness for research in the field of mathematics education is not new, but has existed for a few years. However, a larger effort is necessary for experimental research results to significantly influence users in the educational field. This relationship has been most evident in educational software development. Early mathematical assessment (Van Luit et al., 2015) and math software, are disseminated in the educational literature (Aragón et al., 2013). In these cases, the business interest has been closely linked to the agencies and research group’s prestige. Companies have been able to take advantage of the final product if it has shown efficiency in the applied field. This is the case of ENT, a mathematical evaluation test that has been commercialized by several European companies in the Netherlands (Van Luit and Van de Rijt, 2009), Spain (Van Luit et al., 2015), or Italy (Benvenuto et al., 2018).

But the work that remains to be done in the field of research, development and innovation (R, D&I) of educational psychology is how to involve companies in an initial investment before the development of the research products. In this sense, this paper tries to offer a model of relationship between a company in the children’s entertainment sector and educational research. That is, how to link a company – whose commercial interests need to be realistically understood – with the potential of developing a research project in the educational field. The research project can show corporate profitability expectations in the mid-term. In this sense, a connection has been found between the Babyradio Company ^1^ and our research group’s goals through the design of ICT prototypes.

Babyradio is a radio station company whose contents are broadcast via FM and internet that is focused on entertainment for children from 0 to 6 years old. Its programming reaches 130 countries and more than 350,000 listeners. Among these contents, Babyradio has different children’s characters that act as catalysts for children’s attention through music, entertainment, story, and so forth. The company has highly qualified graphic designers, an R + D + I department. Babyradio’s management team had prior experience in collaboration with research and development projects (Varela, 2014).

The connection of interests between Babyradio and our research group allowed us to develop a set of computer applications for mobile devices (APPs), taking advantage of the synergies between Babyradio’s graphic design team (image and sound) and our prior experience in development of mathematical education software (Navarro et al., 2012).

Consequently, the main goals of this work are threefold: (1) to present the institutional framework of the relationship between the R&D project and Babyradio, a children’s entertainment company; (2) to share the collaboration scheme that may have applied attention for other R&D projects; and (3) to show some of the educational prototypes generated by this research-business collaboration and its applied experimental development.

## Objective #1: R&D Project and Babyradio, a Children’s Entertainment Company Agreement

In a society with a globalized economy, the innovation capacity of research carried out by universities must contribute to their economic and social progress (Mu-Hsuan and Dar-Zen, 2017). This development can be done in different ways: investing in human capital or also strengthening research lines that generate added social value. Universities have as a main focus the generation of innovative and diverse knowledge. To achieve this, one of the possible ways is to establish mutual collaboration relationships with companies. This type of collaboration should be attractive both for the purposes of the company, as for those of the university, its students and its professors.

Although these relationships have to be formally established from the institutional point of view, it must be considered that the innovation parameters and the importance in the generation of socially sustainable knowledge are very significant values to establish these cooperation channels. Likewise, if we want this cooperation between R&D projects and companies to be possible, we must consider realistic timing and companies’ objectives with which strategic agreements are sought.

These agreements should not only be done in the production industry’s field, but also in socio-economic sectors related to services, such as education. In recent years, there has been more interest because education is also a field where entrepreneurship can be promoted, and which can facilitate the making of spin-offs allowing future students to improve their employability. Certainly, the education sector has not been the main focus of the research interest, but a progressively emerging areas of opportunity. An example of this is found in the recent work developed by Fuente et al. (2008), generating a technology-based service company with the aim of influencing the educational field. The spin-off created offers from different professional services based on the results of research, development and innovation projects^2^.

Collaboration between corporations and research has been conceptualized as a complex process. There are different constructs involved such as cooperation, the formation of work teams or the coordination between both groups of interest (Rajalo and Vadi, 2017). We must bear in mind that innovation in the field of research is recognized as both a result and a process. In any case, when it is carried out within the university-company collaboration, it requires taking into account the organizational aspect of the cooperation, as well as its implementation in the field of scientific research. This company’s R&D relationship identifies at least three types of dealings (Rajalo and Vadi, 2017). In the first place, it needs a multidisciplinary perspective capable of focusing the interests of research and the company from different configurations. Likewise, both institutions that collaborate will be immersed in a very rewarding constant process of bilateral learning. And the investment made in both fields should also affect the two sectors involved: the company and the research. This type of relationship must also be evaluated to verify the degree of efficiency and if both institutions achieve the proposed objectives.

Within this general philosophy, we have developed a mutual collaboration agreement between Babyradio Company and the R&D project carried out by the Department of Psychology, University of Cadiz-Spain (UCA). The main objective of this collaboration was to give productive a output to the research results developed since the R&D project, related to mathematical learning for children in early childhood and primary education. The bottom line for this agreement was that, in applied research in the field of educational psychology, the most important goal is that outcomes obtained can be used as broadly as possible within the educational community. It was an important goal that the research generates some socio-educational impact, allowing the investment return that the R&D system has made in the projects.

## Objective #2: To Share Babyradio-R&D Team Collaboration With Other Groups

The relationship between Babyradio and UCA’s research team existed from the beginning of the R&D project drafting. It was an extension of a relationship already established in previous projects. The difference in this case was that Babyradio not only had a role in the dissemination of results, but the company collaborated in the support of graphic design during the initial phase of the development of APP prototypes. In this sense, we incorporated into the computer design image and sound resources provided by the design team made by the company’s staff. The flow of information between Babyradio’s staff and research team involved in software design was continuous, thus guaranteeing the generation of synergies between the two groups.

The children’s entertainment industry is highly developed by large multinational companies, and there is a company concentration process so that the culture and educational outcomes are also under the effects of globalization (Noam, 2016). Small companies in this area need to make a considerable effort to stay in this difficult market, dominated by large service platforms. One of the ways to remain in this business is seeking agreements with institutions that provide added value to the contents and services offered to potential consumers. This extra value can generate innovative outcomes, contrasted in educational settings. This is how the mutual interest of collaboration between educational research and certain companies in this business sector should arise.

**FIGURE 1:**
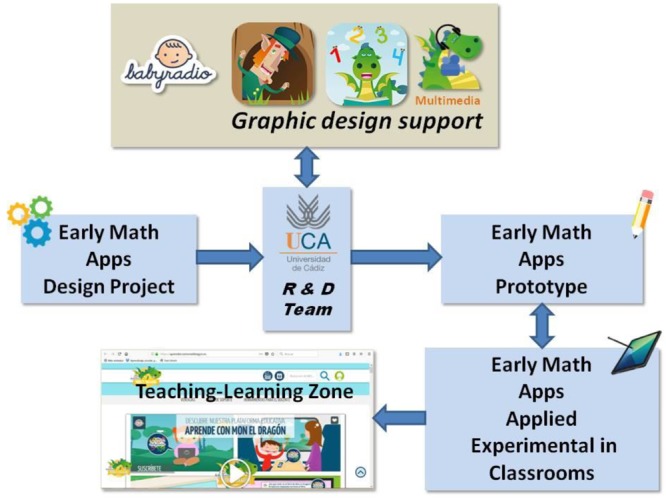
Structure of interchange R&D project and Babyradio, a children entertainment company.

The Babyradio-R&D team collaboration chart summarizes the structure (Figure 1). However, considering the differences in timing, resources and hierarchy of decision making between the business and research areas, we surmised that there were three requirements that had to be guaranteed for the success of the R&D-company relationship: (1) establish an official collaboration within the framework of university-company institutional relationships; (2) unequivocally decide the specific human resources that both Babyradio and the R&D team would establish for the flow of technical information; and (3) unambiguously specify the exchange of technical resources (graphics, formats, multimedia resources), as well as convenient deadlines for both parties.

In this sense, the first level of the agreement (Figure 1: early math APPs design project) was reached between the staff in charge of the R&D team and Babyradio’s executive management. The second level would be a more technical collaboration between those responsible for the software design of the R&D team and the link designer provided by Babyradio. Finally, the third level was the prototypes’ implementation in educational settings (Figure 1: early math applied experimental in classrooms) and dissemination on Babyradio’s website, with the commercial conditions that the company understands are more advantageous.

However, this collaboration agreement’s approach would not be possible without contrasted results and with an appropriate timing for the two entities. In this sense, the outcomes generated by the R&D project have consisted of several APPs prototypes, of which we will describe only four, for practical reasons. These APPs deal with content about early mathematics, in a field applied education, which we describe briefly below.

## Objective #3: Apps Prototypes Description and Implementation in the School Setting

Currently, children are immersed in an environment in which mobile devices have become the most widespread resources, in terms of communication and leisure (Livingstone et al., 2018). The design of the APPs arises from the existing need in educational settings to provide new methods of training. These should be playful and adapted to young children’s everyday life. That was the target population for this project. Previous work has highlighted the advantages of the use of technological tools in teaching in general (Herodotou, 2017), showing explicitly positive effects in mathematics (Baker et al., 2018).

### Prototypes Description

#### Prototype Description: Comparison of Magnitudes Task: Compare Amounts With Mon the Dragon

The main target of this APP was the training in comparison of magnitudes, one of the specific domain skills predicting high achievement in early mathematical learning (Aragón et al., 2016; Cerda et al., 2017).

**FIGURE 2:**
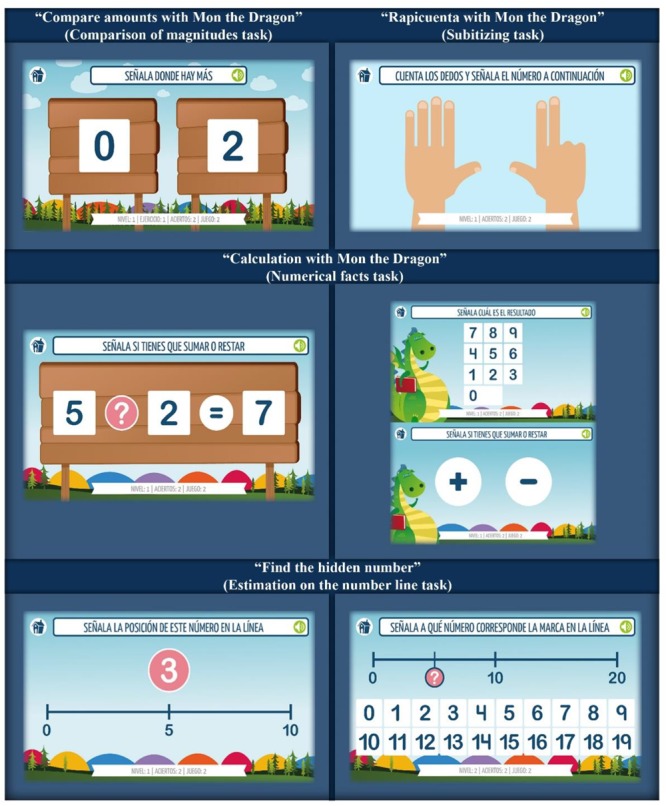
APP prototype screenshots for several mathematical tasks.

This APP presents a game which consists in identifying and indicating which amount is higher or lower between two response options, as indicated in the statement (Figure 2: “Compare amounts with Mon the Dragon”). These two alternatives can be displayed in the same format (symbolic or non-symbolic), or be presented in a mixed style. After the initial screen, a menu with three levels of difficulty appears. These levels have implicit sub-levels that grade the game complexity. Each exercise offers 10 attempts in which the player must point out where there is more or less. To overcome the level, participant must correctly answer 80 percent of the attempts.

#### Prototype Description: Subitizing Task: “Quick Counting With Mon the Dragon”

The goal of this task is the subitizing training (sudden counting). The additional goal is also improving the numerical sense. According to the design, the knowledge of the numerical line should also be enhanced. The player must count a number of elements in a short period of time (4 s), with different levels of difficulty. Two screens appear on each task: (1) Stimulus-screen with verbal and written instruction (Figure 2: “Quick counting with Mon the Dragon”); and (2) Answer-screen with a number line for pointing the response of the sudden count. The APP has 10 items per level. To overcome this, it is necessary to get an 80 percent correct rate. After the response, feedback is given. If there is no response within 10 s, the item is registered as incorrect.

#### Prototype Description: Numerical Facts Task: Calculation With Mon the Dragon

Numerical facts correspond to simple calculations stored in long-term memory (addition, subtraction, and multiplication or division). These are useful features in understanding and developing arithmetic concepts, facilitating the problems solving. During this APP game, the child must solve different calculations presented (addition or subtraction). On other tasks, the unknown is not the result, but some element that makes up the operation (addendums or subtracts), or the sign of the numerical facts. Here, the child must identify if it is a sum or a subtraction (Figure 2: “Calculation with Mon the Dragon”).

Each item or operation that must be solved appears for 4 s. After that time, the response screen is displayed, which will remain until the child points with his finger a numberor chooses a sign (Figure 2), depending on the question and level of difficulty.

#### Prototype Description: Estimation on the Number Line Task: Find the Hidden Number

The purpose of this APP is to extend the knowledge of numbers and their position in a straight line. The estimation task is considered as a specific domain cognitive precursor relevant for improving mathematical skills such as counting, arithmetic operations or the understanding of mathematical concepts. The domain of the number line allows the child to answer questions regarding the magnitude without referring to specific objects; this supports the cardinality’s modification rule of a set, depending on the addition or subtraction, and allows them to know the relative position of a number when the task cannot be directly solved (Gervasoni, 2005; Siegler and Booth, 2005).

Two types of estimation coexist in this APP (Figure 2: “Find the hidden number”). In one case, a number appears, then the child must place it in the appropriate position in a straight line (number-position); or the straight line has a distinctive mark in a specific place. Then the child must decide to which number that place belongs (position-number). A video tutorial has also been created because of the difficulty in understanding the task. The video tutorial is available from the initial screen. Each level presents 10 activities. Users have to score at least 80 percent to get to the next level. The correction of the activity is through an approximate error procedure, developed according to the following algorithm: {(X–Y)/Z}× 100. Where X, number requested; Y, number that the child indicates on the number line; Z, size of the line. The margin of success is considered as a 7.5 percent deviation from the correct answer.

#### Technical Design of Prototypes

The development of the APPs interactive section on mathematical content is based on the current Web technology: HTML5, CSS, and JavaScript. The standardization of web browsers in recent years has made JavaScript one of the most important programming languages. JavaScript is an interpreted language. So, each time a program or script runs, it must be translated into recognizable codes by the microprocessor. These programs are less efficient than those developed with compilers, in which the code once translated or compiled is stored in one or more files and executed without any conversion. The great advantage of JavaScript is that its interpreter is incorporated into the Web browsers and specifically translated by the platform’s operating system on which it is running. In this way, a single development that meets the language standards can run in any browser of any platform and operating system that follows these standards.

Based on this standardization, hundreds of work frameworks based on JavaScript have been developed. They generally are dedicated to very specific activities within the software programming. The CreateJS framework^3^ is modeled on the Adobe Flash author software with which we have developed several educational software in the past. Starting from this knowledge, we are granted many advantages, facilitating and accelerating our developments, mainly in the management of images, animations and sounds, which make up the main core of the APPs we developed. However, CreateJS does not provide appropriate solutions to the treatment of form-sheets – essential for the data’s control and storage. To solve this, we also used the jQuery Mobile framework^4^. This software contains jQuery framework^5^.

**FIGURE 3:**
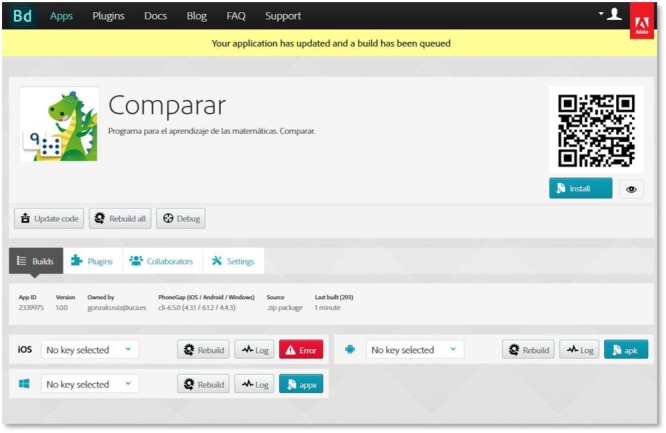
Interface screenshot for Phonegap Build.

Another consequence of JavaScript standardization, along with HTML5 and CSS3, is the appearance of frameworks for the conversion of Web applications (created with these languages) into fully functional APPs for mobile devices of the most widespread platforms. Apache Cordova is a free software solution (open source) for the recognition of these conversion tasks to APPs. In our developments, we use Adobe Phonegap, a distribution by Apache Cordova. One of the built-in elements is Adobe Phonegap Build (see Figure 3), an online application that directly allows APPs packaging in the cloud, facilitating the conversion process. This application is 100 percent available for Adobe Creative Cloud subscribers. With Phonegap Build, in a simple way, we managed to expand the distribution of our applications to a wide collection of devices, such as smartphones and tablets, based on a single development and almost without changes.

In order to avoid repeatedly entering the data into the form-sheets, we used the local storage resources provided by HTML5 (local storage), known as Web storage. When working under conditions in which an Internet connection is not available, the data generated during the execution of the APPs are locally stored, using SQL Web data. In this way, all data stored in the device can be synchronized with a remote database. In the Web format, this decision limits the use of some browsers when we process data because not all of them support Web SQL. Chrome, Opera and Safari support it perfectly, but not Mozilla Firefox or Microsoft browsers.

The remote data storage was made in relational databases with the MyQSLi interface (MySQL Improvement extension), using Ajax and PHP as languages for connection and data management. The jQuery framework facilitates asynchronous transfers, through Ajax, between the local and the remote database.

##### Media resources

The graphic pieces of the application have been designed in Adobe Illustrator. Therefore, they were originally of a vectorial format. The complexity of some of the designs, in terms of elements and color gradients, such as the background or pet (*Mon the Dragon*), forced us to convert them to a bitmaps format, specifically to PNG files to keep transparencies. With Adobe Photoshop, we adjusted these bitmaps and calculated sizes and proportions so that everything could take its place according to the initial design.

Easier elements, such as buttons, icons, dice, and so forth were converted using Draw2script – a plugin from Adobe Illustrator – into a collection of graphic commands used by CreateJS (EaselJS Tiny Api). This allowed us to draw all components in real time on the screen. The simplest ones were drawn using the basic CreateJS commands.

The integration of all these features was done through CreateJS, so that everything could take its place and be observed by the user as a single design. Also, with CreateJS, we synchronized all sounds. We used Audacity and Adobe Audition software. All the sounds were stored in mp3 files.

### APPs Prototypes Implementation in School Settings

To implement the project, cognitive variables of general and specific features involved in early mathematical learning were selected. Then all activities were planned in a game context, emphasizing the process of gamification, in order to obtain positive results based on their advantages (Hanus and Fox, 2015; Papadakis et al., 2018). Subsequently, Babyradio focused on the APPs’ design improvement, adding voices, graphics and animations that were processed by researchers. The APPs’ prototypes implementation allowed us to validate, with a significant sample, APPs’ usefulness in training those mathematical skills established by the designers.

#### Experimental Procedure

A quasi-experimental design with pre- and post-intervention measures was carried out. A quasi-experimental design was used. This distribution was based on the participant’s mathematical achievement and some ecological issues, such as the group-classroom and the school characteristics.

#### Participants

For APP validation, a sample of 112 students of the last year of early childhood education (5 years old) was used. Participants were aged between 58 and 79 months (*M* = 63.45, *SD* = 3.46). Considering participants’ gender, 60 were girl between 58 and 80 months (*M* = 63.37, *SD* = 4.42); and 52 were boys aged between 58 and 71 months (*M* = 63.54, *SD* = 3.55). The children with special educational needs capable of completing the evaluation batteries and the intervention program were included. They were assessed by the pre-tests and by teachers’ and the school technical team’s expert judgment. In total, of the 118 students that were part of the initial group, six students were diagnosed as special needs students. This study was carried out in accordance with the recommendations of the Bioethics Committee of University of Cádiz. All subjects gave written informed consent in accordance with the Declaration of Helsinki and Singapore Statement.

#### Evaluation Instruments

Different evaluation tests were used for participants’ assessment of cognitive and numerical knowledge, in order to validate the APPs (Table 1). The different tests allowed us to know both the domain of numerical knowledge, as well as the general and specific cognitive variables related to early mathematical learning^6^.

**Table 1:** Evaluation tests used in the study for APP prototypes validation of improvement of early mathematical competence.

General factors	Specific factors
Symbol search task, from The Wechsler Preschool and Primary Scale of Intelligence WPPSI-III (Wechsler, 2009) (Cronbach’s *alpha* 0.84)	Test of early mathematics ability TEMA-3 (Ginsburg et al., 2007) (Cronbach’s *alpha* 0.91)
Test receptive vocabulary test task, from DSJ-T (Fawcett and Nicholson, 2013) (Cronbach’s *alpha* 0.74)	Estimation task (Siegler and Booth, 2005) (Cronbach’s *alpha* 0.80)
Forward and backward digit span tasks, from WISC-IV (Wechsler, 2005) (Cronbach’s *alpha* 0.76 and 0.78)	Symbolic and non-symbolic comparison task (Nosworthy et al., 2013) (Cronbach’s *alpha* 0.82)
Short-term memory and working memory test (Lanfranchi et al., 2004) (Cronbach’s *alpha* from 0.70 to 0.88)	

#### Procedure

First, a pre-test evaluation was carried out in two sessions for all participants. In one of the sessions, students individually completed specific tasks related to their early math skills. The second session was focused on assessment of cognitive parameters related to mathematical learning. Sessions were randomly run on different days. Within the sessions, the tests were randomly applied to control any effect of fatigue or facilitation in the variables measurement.

Once the total number of students was evaluated, those children belonging to the group that should receive the intervention training were selected. This selection was carried out based on the hypotheses that were intended to highlight the effectiveness of the use derived from APPs for low- and high-math-performance children. Consequently, the student’s score was considered and compared with peers from their same group by selecting the three students (above the 75th percentile) with the best math skills, and the seven students who scored the lowest (below the 50th percentile) in the specific test of mathematical evaluation. This distribution was made according to the ecological characteristics of the groups (classroom grade, teachers, etc.,...).

Once the experimental and control groups were selected, the intervention was started. A total of 30 sessions were planned: three (3) sessions per week, of the duration of 30–35 min per session, in groups of 10 participants per session. All sessions were conducted with the supervision and guidance of specially trained professionals. The students worked with mobile devices (tablets and smartphones randomly). They used headphones to facilitate concentration on tasks. Once the intervention phase was finished, all students were again evaluated on the same cognitive and mathematic variables during two post-test sessions in order to judge the effectiveness of the intervention through APPs.

#### Results

Considering that the main goal of this article was to describe the institutional framework of the relationship of the R&D project and a children’s entertainment company, a short result report is presented. This exploratory study presents information on the descriptive statistics of the evaluated variables (Table 2).

**Table 2:** Descriptive data of the experimental and control groups.

		Pre-test		Post-test	
Groups		Mean	*SD*	Mean	*SD*
Control *n* = 62	Informal conceptual mathematical thinking	19.61	2.730	24.76	3.337
	Formal conceptual mathematical thinking	3.27	0.944	4.42	1.262
	Total	22.56	3.055	29.18	4.430
Experimental 1 (Low performance) *n* = 35	Informal conceptual mathematical thinking	13.77	2.414	23.49	3.100
	Formal conceptual mathematical thinking	2.23	0.731	4.57	1.836
	Total	16.00	2.787	27.97	4.643
Experimental 2 (High performance) *n* = 15	Informal conceptual mathematical thinking	26.13	3.563	32.20	2.242
	Formal conceptual mathematical thinking	5.20	1.612	10.00	3.742
	Total	31.33	5.024	42.20	5.570

Both the students with low performances (experimental group 1) in mathematical competence and risk of dyscalculia, and the students with high mathematical performances (experimental group 2), increased the scores in each of the mathematical subtests evaluated. The experimental group 1, of low mathematical performance, was compared to the control group in all the mathematical records. The differences obtained with respect to the control group were statistically significant for experimental group 1 (*t*_61_ = -14,093; *p* < 0.001) and for experimental group 2 (*t*_49_ = -19,606; *p* < 0.001). The results could be explained by the effectiveness of the APP training designed to work on the specific predictors of mathematical learning. However a total comparison analysis is necessary.

## Limitations and Conclusion

The main objectives of this study were to describe the institutional framework of the relationship of the R&D project and a children’s entertainment company (Babyradio); to share the collaboration scheme between the working groups of both levels; and to show some of the applied educational outcomes generated by this collaboration. It was not the goal of this study to show specific data analysis about the APPs’ effectiveness in the field of early math learning. The study is based on the collaboration agreement between a children’s entertainment company and a university’s research group. Although the experience was highly positive and productive, the conclusions must be considered, taking into account the context where it has been developed. Therefore, a generalization for cooperation agreements with any type of company R&D must be made with caution. The dynamics established between the work teams of both parties should be conveniently handled, with the least possible interference. The interest and the exchange of qualified technical information must prevail over any other information (economic, organizational, etc.) that may contaminate the objectives of the relationship. Although the empirical study still requires a multilayered data analysis, there are some practical recommendations that can be suggested. Mathematical learning is critical for an adequate school and social adaptation. The strategies used are numerous but they should be aimed at intensifying the learner’s cognitive resources. Those teaching-learning approaches that help improve the general and specific cognitive precursors of mathematics should be implemented efficiently with young children. The APP designed in this study should be one of the useful strategies to reduce the risk of mathematical difficulties in preschooler. Given the limitations of our study, a more extensive analysis of other entrepreneurship experience in the field of educational psychology would be required, with a longitudinal methodology. When strategies of entrepreneurship education are discussed, some attention should be paid to stakeholders’ affinity and embeddedness within university-industry cooperation framework. Future research should spread our awareness of the role of innovative education in academic spin-off, employability and make-bring-returns of knowledge. This methodology would allow us to verify the advantages of the need for a multidisciplinary perspective to focus on the research interests and of the company; and to, therefore, ascertain whether profitable bilateral learning has occurred and if the investment in human capital has been productive for both parties.

## Author Contributions

CM, GR, MA, EA, CD, IM, EM, MGS, and JN designed, evaluated, and wrote the manuscript.

## Conflict of Interest Statement

The authors declare that the research was conducted in the absence of any commercial or financial relationships that could be construed as a potential conflict of interest.

## Footnotes

1 www.babyradio.es

2 Website of a spin-off developed by a University of Almería (Spain) project:http://www.education-psychology.com/ebt/new/espanol/index.php

3 https://createjs.com/

4 http://jquerymobile.com/

5 http://jquery.com/

6 It was not the goal of this study to show specific data analysis about the APPs’ effectiveness in the field of early math learning. For that reason, just a brief summary of the evaluation instruments is presented.
